# Impact of Live Preferential Music on Pain in the Emergency Care Setting: A Randomized Controlled Trial

**DOI:** 10.5811/westjem.61331

**Published:** 2026-09-09

**Authors:** Jill Sonke, Marie-Carmelle Elie, Diana J. Wilkie, Henry W. Young, Muhammad Abdul ‘Baker’ Chowdhury, Meenakshi Puthucode Balakrishnan, Cindy Montero, Joseph “Adrian” Tyndall

**Affiliations:** * University of Florida Center for Arts in Medicine, Gainesville, Florida; † University of Alabama at Birmingham Heersink School of Medicine, Department of Emergency Medicine, Birmingham, Alabama; ‡ University of Florida, College of Nursing, Department of Biobehavioral Nursing, Gainesville, Florida; § The Ohio State University, College of Medicine, Department of Medicine, Emergency Medicine, Columbus, Ohio; ¶ University of Florida Institutional Review Board, Gainesville, Florida; || University of Florida, Department of Neurosurgery, Gainesville, Florida; # IQVIA, Newberry, Florida; ** Western Michigan University Homer Stryker M.D. School of Medicine, Kalamazoo, Michigan

## Abstract

**Introduction:**

Pain is the most prevalent complaint for the more than 155 million patients accessing emergency care in the United States. Appropriate and timely management of pain is a measure of quality emergency care. However, the opioid crisis has created a need for multimodal and alternative approaches to successful pain management. Models for integrating such approaches in emergency departments (ED) are understudied. Therefore, we chose to investigate the impact of live preferential music (LPM) in the management of acute musculoskeletal pain.

**Methods:**

This prospective, randomized, double-blind intervention study was designed with block randomization clustered by day of the week to live preferred music versus usual care. To ensure blinding, patients were deceived that the study focus was on pain assessment without disclosure that there was an intervention. A total of 2,262 patients were screened, and 272 patients (94 interventions, 128 usual care controls, and 50 declined-music intervention) were recruited from two free-standing EDs. All subjects received analgesics per prescription, had vital signs measured, and completed baseline and follow-up Pain Intensity Number Scale (0–10) scores via PAIN*Report*It. We calculated morphine equivalents for pain medications in the ED.

**Results:**

The mean age of the 222 patients in the per-protocol analytic sample was 48.3 years (95% confidence interval, 45.9–50.7); 64% were female, 29% Black; and their triage acuity was 4% emergent Emergency Severity Index (ESI) 2, 52% urgent ESI 3, and 44% less urgent ESI 4. There were absolute reductions in current pain intensity from baseline in both the control (−1.21) and intervention (−1.34) groups (difference −0.14, 95% CI, −0.48 to 0.76). However, the difference was not significant in posttest pain intensity when controlling for baseline pain intensity (*P* = .29). Qualitative analysis reflected patient-reported reduction in the perception of pain as a common theme. Vital signs and morphine equivalents were similar between groups.

**Conclusion:**

This study demonstrated that live preferential music interventions are feasible to study rigorously in the ED. Patients were enrolled using deception and were not informed of the study’s focus on music or their potential exposure to music. However, they were told initially that they would be part of a pain assessment study. Although significant between-group differences were not observed, the general theme of patient-reported reduction in the perception of pain suggests that a more highly powered study might detect significant effects in pain reduction with LPM. Variation in clinician care strategies and evolving federal guidelines for pain management may have influenced study processes and outcomes.

## INTRODUCTION

For the more than 155 million patients accessing emergency care in America every year, pain is the most prevalent complaint.^1^ Although failure to recognize and treat pain in an appropriate and timely manner is a marker for poor emergency care, rise of the opioid crisis underscores the need for multimodal and alternative approaches to successful pain management. Models for integrating such approaches in emergency departments (ED) are understudied. This study investigated the effects of live preferential music (LPM) in an emergency and trauma care setting on acute musculoskeletal pain intensity and medication administration.

Emergency departments are high-stress environments that are one of the significant drivers of high costs in healthcare. Due to the relatively high pace, crowding, and resource-intensive environment, compared to other clinical settings, the ED has traditionally proven to be a challenging setting for the delivery of interventions including music. Although music therapy has been shown to reduce pain and anxiety in emergency settings,^2^ LPM—patient-selected songs performed vocally with instrumental accompaniment—has been studied primarily outside acute care,^3^,^4^ and remains largely unexplored in emergency and trauma settings.

This study was grounded in neuroscience evidence that music activates the brain’s reward system, including dopamine release, and that both familiar stimuli and the act of choice are neurologically reinforcing.^5^ Furthermore, the trial was supported by experience of two prior unpublished protocol development studies at the University of Florida (UF) and UF Health. The first study focused on the development of strategies for introducing LPM in ED settings, and the second unblinded study focused on the feasibility and impacts of this intervention in a primary hospital-based ED where most patients were exposed to two to four songs. In this third study, we aimed primarily to determine whether LPM could reduce pain intensity and secondary reduction in pain medications administered for patients presenting to the ED with musculoskeletal pain. We also examined the physiological effects of LPM on patients’ vital signs, blood pressure, heart rate, respiratory rate, and oxygen saturation. Finally, we examined the fidelity of implementing a LPM protocol for obtaining specific preference-based musical requests from patients in the ED.

## METHODS

### Study Design

This prospective experimental trial, conducted with a convergent mixed-methods design, included a randomized, controlled, double-blinded design with one experimental and one control arm to assess the effects of a new LPM protocol (ClinicalTrials.gov NCTT03327077). The University of Florida’s Institutional Review Board (IRB201700938) approved the study.

A block randomization clustered by day of the week to LPM or usual care was used to determine the days of music intervention over the duration of the study. Within blocks of four, six, and eight days, randomly ordered, the days were assigned to music intervention or no music intervention (usual care). Patients were enrolled using deception and were not informed of the study’s focus on music, or their potential exposure to music; however, they were told initially that they would be part of a pain assessment study. After the study was completed, we mailed letters to participating patients disclosing the deception.

**Population Health Research Capsule**

What do we already know about this issue?

*Pain is the top emergency department (ED) complaint; opioid limits heighten interest in nonpharmacologic options, but ED music models remain understudied*.

What was the research question?

*Does live preferential music reduce acute musculoskeletal pain in ED patients compared with usual care?*

What was the major finding of the study?

*Post-music pain scores declined over time but were not different from usual care: Pain score decline: −1.21/10 control vs −1.34/10 music; difference −0.14 (95% CI, −0.48 to 0.76)*, P *= .67*.

How does this improve population health?

*These findings support feasible, acceptable nonopioid ED pain strategies that may improve patient-centered care and warrant larger trials*.

### Sample Size

Trained research associates (RA) recruited patients from those seeking care in two free-standing EDs. Inclusion criteria included English-speaking patients 18 years of age and older with a chief complaint of musculoskeletal pain, grade two reading level or greater, and the capacity to participate physically and cognitively. Patients who could not consent and prisoners were excluded. Based on study duration and clinical feasibility for detection of a 25% reduction in pain with 80% power, a total of 242 subjects (121 per group) was required.

### Procedures

The study took place with trained musicians and RAs in 5-hour shifts from 12 pm – 5 pm on randomized weekdays. Only the study statistician and the assistant director of the Arts in Medicine program, who was responsible for supervision of the musicians, knew the days assigned to each condition. The RA and musicians were made aware of the music day assignment on the date of randomization. On all study days, patients were screened for participation and enrolled in the study via written informed consent by a RA in the ED. The RA then administered the prequestionnaire and on music days notified the musician of the patients who consented. The musician subsequently consulted with the charge nurse and/or attending physician(s) to identify patients for whom music would be appropriate. If the clinician agreed, the musician entered the patient’s room and offered music to the patient within a 15-minute window of completion of the premeasure.

If the patient was interested, the musician discussed music preference with the patient using the LPM protocol (Supplemental Materials) and offered to perform appropriate preference-based music. For all enrolled patients, the RA returned to the patient room 30 minutes after the completion of the premeasure to obtain the postmeasure. This period allowed a standard time frame for both groups with wait time for the control group and sufficient time for the music intervention.

### Usual Care

Patients consenting on days randomized to usual care received prescribed analgesics. They completed the study procedures (ie, pre- and post-measures and vital signs).

### Music Intervention

Patients consenting on days randomized to the music intervention received usual care (prescribed analgesics) and study procedures (ie, pre- and post-measures and vital signs) and after the RA exited the room, the musician entered and offered to perform for the patient; the patient did not know that music was part of the study. The RA was not informed whether the patient accepted the music; all patients enrolled on days randomized to the music intervention completed all study measures.

Per the protocol, each conversation between the musician and patient, following introductions, started with the question “What would you like to hear?” and subsequently went from broader levels of preference (genre) to specific (song), based on whether the patient had an initial music preference or not. The protocol had six distinct pathways to preference. Musicians used the protocol as a framework, rather than a script, and always deferred to the patient’s comfort with answering questions.

Musicians were guitarist-vocalists with broad musical repertoires that allowed them to respond to a range of requests across musical genres. The patient’s door remained closed during the interaction to limit the drift of sound to other areas. Per the normal practice of artists in the hospital’s Arts in Medicine program, patients were provided the option to decline or discontinue the interaction at any time.

To assess the intervention processes, the musicians documented their interactions with patients on a Research Electronic Data Capture (REDCap) form after each performance. The form documented the location, time and length of the interaction, the number, and roles of the people in the room, the patients’ sex, the music played (genres, artists, songs), and how preference was (or was not) established in relation to the LPM protocol. Protocol fidelity was examined through analysis of the pathways taken to obtain preference. These pathways were mapped using an online Qualtrics LLC survey that documented the musician’s questions and patient’s answers regarding musical preference. The objective was to garner preference using the fewest questions possible. The musicians also entered a narrative description of the interaction.

### Outcomes and Measurement

The primary outcome of this study was the assessment of pain reduction using PAINR*eport*It^6^ collected via Apple iPad or Microsoft Surface devices. PAINR*eport*It is a validated computerized extension of Melzack’s (1970) McGill Pain Questionnaire (MPQ).^7^ Its Pain Intensity Number Scale (PINS)^8^ allows subjects to indicate the intensity of pain at present, as well as their least and worst pain during the prior 24 hours (baseline) or since the last measure (postintervention). The PINS provides ratio-level data as a measure of pain intensity^9^ with its scale of 0 (“no pain”) to 10 (“pain as bad as it could be.” Concurrent (r =.80–.89)^8^ and construct validity have been reported.^10^,^11^ The PINS with its standardized instructions can be completed by patients in less than one minute.^8^

Secondary effects of LPM included morphine equivalents and physiological measures. Variables were collected and cross-referenced using the subject’s medical record number and the date of visit. Bedside collection of the patient medical record number, music selection, and date/time of the intervention were recorded at the time of the musician visit. Variables collected from the medical record included patient age, sex, mailing address, blood pressure, heart rate, oxygen saturation, and respiratory rate before and after the music intervention. All data were entered into the university’s secure REDCap database.

### Statistical Analysis

We exported pain data from the PAIN*Report*It structured query language (SQL) database into Microsoft Excel and imported into Statistical Package for the Social Sciences (SPSS25) for statistical analysis. All clinical data were extracted from the REDCap to SPSS. Live preferential music protocol data were analyzed using SAS Institute Inc. Descriptive statistics (means, frequencies, and percentages) and inferential tests (the independent *t* test, chi-square test, and Fisher exact test) were used to examine the relationships between the outcome variable and the covariates. All *P* values were from two-sided tests, and results were deemed statistically significant at *P* < .05.

We chose a per-protocol analysis approach over an intention-to-treat approach because patients were unaware that the intervention was music-based, having been told the study’s sole purpose was pain assessment. The primary study outcome was the between-group difference in post-pain intensity scores after the group condition (wait period or music intervention), controlling for the baseline pain intensity score. The model for this was as follows: postintervention pain score = constant + (*a* × baseline pa*i*n score) + (*b* × group), where *a* and *b* are regression coefficients and group is a binary dummy variable (with the control coded as 0 and treatment coded as 1). The main coefficient of interest was *b*, which was the estimated difference between the treatment and control group. An analysis of covariance (ANCOVA) adjusted each subject’s follow-up score for their own baseline score but was unaffected by baseline differences and regression to the mean. Statistical significance a priori was set as *P* < .05.

Qualitative data garnered from the musicians’ narrative reports following each music interaction and decline were analyzed thematically by a team of investigators and RAs. A codebook was developed to define and document themes. The resulting themes were used to explain and triangulate with the quantitative findings. Assessment of the fidelity of the LPM protocol was undertaken through quantitative analysis of the pathways the musicians took to obtain musical preference from patients.

## RESULTS

### Sample Characteristics

A total of 2,262 patients were screened for the study, and 272 patients were consented to participate at two free-standing EDs. Of those who consented, 94 received the music intervention and 128 received usual care in the control group. An additional 50 patients were consented on intervention days and completed measures but declined the music intervention and were subsequently excluded from the main analysis, which included 222 total patients (Figure).

Table 1 outlines patient characteristics as well as differences in characteristics between the control and intervention group. The mean age of the sample was 48 (95% CI, 46–51); age ranged from 18 to 95 years. Most patients were female (64%) and White (71%), and 37% were privately insured. The triage acuity distribution was 4% emergent ESI 2, 52% urgent ESI 3, and 44% less urgent ESI 4. The mean body mass index was 31.3 kg/m^2^ and mean length of stay was 4.4 hours (95% CI, 3.78–5.03). As shown in Table 1, the control and intervention groups did not differ significantly on any of the measured personal or clinical characteristics (*P* > .05).

### Outcomes

Table 2 shows the mean pain intensity score of all three pain measurements by groups and changes in pain intensity scores within group. The *pain now* had the highest reduction of pain intensity for the control (−1.21) and the intervention (−1.34) groups among the three pain measurements.

Table 3 shows the results of the ANCOVA model, in which we adjusted for the baseline pain score effect on the postintervention pain score. Results show that although there were absolute reductions in pain intensity for patients who were randomized to control or intervention groups, the differences in the pain intensity were not statistically significantly different between-groups (*P* > .29). Absolute reductions in pain scores were more clinically significant in patients with moderate initial pain intensity scores as opposed to higher or lower pain intensity scores. We conducted a similar analysis—adjusting for patient’s sex, age groups (younger than 50 versus older than 50 years), race, and payer status—and found no significant predictor except baseline pain score.

There were no differences in vital signs (Table 4) or pain medication administration between groups as determined by morphine equivalent administration (Table 1).

### Qualitative Findings

The qualitative data garnered from musician’s narrative reports resulted in seven themes (Table 5). It was notable in the qualitative analysis that all the themes were positive in nature, generally and overtly reflecting appreciation and satisfaction with the music intervention. A common theme was patient-reported reduction in the perception of pain, suggesting that a more highly powered study may detect more significant effects on pain.

### Live Preferential Music Protocol Fidelity

Among the 94 participants in the music intervention group, six were missing data and nine noted “other” as a means of achieving preference. These 15 records were removed from analysis, leaving 79 LPM data points. Initial preference was documented for 49 patients (62%), whereas 30 (38%) did not express an initial preference. When patients had an initial music preference, P3 (genre–artist–song) was the most common pathway to preference (77.5%). When patients did not have an initial music preference, NP3 (tempo–genre–artist–song) was the most common pathway (70%). Across both initial and no initial preference groups, the most prevalent ending point in the dialogue for determining musical preference was genre (44.3%) followed by tempo (22.8%). Compared to earlier study phases, which were only able to obtain clear preference from 49% of patients, the protocol was determined to be an effective and efficient means for determining preference, as it resulted in preference being obtained and documented for 100% of patients at the level of genre, tempo, artist, or song.

The music interactions lasted 8–9 minutes, including conversation and performance. Among patients who enrolled in the intervention group, most were only exposed to one song (53%) or two songs (36%).

### Financial Cost Estimates

For our program, we invested in an initial 12-month start-up period to develop and refine practice and protocols. The contracted musicians were paid $30 per hour. Following an initial start-up investment of $23,700, the program was maintained at a cost of $87,000 per year, including hourly pay for the musicians (with 30 total contact hours per week), a part-time coordinator, two RAs and a statistician, and additional in-kind support for supervision by leaders of the hospital art program and research effort by the study co-investigators. The total annual cost for the study, including in-kind support, was $143,700 per year with $63,000 for music program costs and $80,700 in research costs.

### Challenges

There were notable challenges in the initial execution of the study. Enrollment was initiated during the respiratory viral season with unexpectedly high patient volumes. Consequently, access to acoustically isolated areas to facilitate the music intervention was limited. In addition, masking posed challenges in communication between the musician and patient. Therefore, the music protocol was temporarily suspended at a large academic ED and relocated to two satellite or free-standing EDs, which were less prone to crowding during the proposed period of the intervention. Despite this effort, the study team encountered protracted enrollment opportunities, secondary to the lower acuity and shorter length of stay of the free-standing population.

## DISCUSSION

We examined the effects of live music for patients seeking ED care for musculoskeletal (MSK) pain, a common reason patients seek emergency care. We demonstrated that LPM intervention can be rigorously studied in an ED environment, was well-received by the patients, but did not show a statistically significant benefit on MSK pain outcomes or other secondary outcomes. Reasons for the not statistically significant findings are not clear from the data we collected but could include the smaller sample than planned for adequate power, relatively short intervention sessions, and variation in emergency clinician care strategies with the institution of new state and federal pain management guidelines.

Improving quality of care for MSK pain is an important priority for patients, clinicians, and policymakers. Musculoskeletal pain conditions (eg, low back pain, osteoarthritis, cervical and thoracic spine pain) share similarities in mechanisms, prognosis, and clinical trajectory, which has led to overarching clinical practice guidelines for assessment and management.^12^ Recommendations include patient-centered care with effective communication, shared decision-making, screening for serious pathology, physical examination, and assessment of psychosocial status. Given the current opioid public health crisis, nonpharmacologic therapies, including those that promote relaxation, such as live music, are becoming important in reducing the dependency on opioid prescription therapy and could be an important adjuvant to other pain reduction strategies. Reduction of pain by 1 to 2 points on a 0 to 10 pain intensity scale or a 30% reduction is typically considered clinically important,^13^ and patients greatly appreciated the relief, especially if severe pain (7 to 10/10) is reduced to moderate (4 to 6/10) or mild (1 to 3/10) pain.^14^ In our study, we observed a reduction of more than 1 point in both groups treated with prescription medications in both groups and combined with LPM in the experimental group.

Over the past two decades, partnerships between musicians, music therapists, and clinical researchers have yielded unprecedented development of clinical interventions supported by rigorous scientific studies.^2^,^15^ Hundreds of studies have confirmed the effect of music on reducing pain, anxiety, and other clinical measures such as vital signs.^16^ There is clearly documented evidence of the physiologic impact of music including effects on parasympathetic activity, stress hormone levels, and immunity, suggesting its efficacy in decreasing stress-induced autonomic and neuroendocrine arousal and the facilitation of the relaxation response. In the medical environment, music can be a safe and low-cost, nonpharmacologic intervention to reduce anxiety and enhance relaxation in intensive care patients as well as to reduce pain perception, anxiety, and stress levels in the ED setting.^17^

In various patient populations and procedures, music positively affects pain control, pain tolerance, and pain perception,^18^–^24^ as well as the need for anesthesia and sedation.^25^,^26^ The 2008 analysis by Nilsson^27^ of 42 studies found that approximately half of the studies reported significant positive effects of music on pain and anxiety. The 2016 meta-analysis by Lee^28^ of 97 studies found that music interventions had statistically significant effects in decreasing pain, emotional distress from pain, opioid intake, nonopioid intake, systolic blood pressure, diastolic blood pressure, and respiration rate. Also, studies of music in emergency care have presented positive outcomes related to anxiety and stress,^29^,^30^ pain management,^30^–^32^ reduction of noise stress,^29^ and significant pain and anxiety reduction during burn dressing changes.^33^,^34^ Most interventions used recorded music, and some engaged technology such as iPods^35^ in contrast to our use of LPM.

In our study, participants were blind to the study goals, and our findings add to the few unblinded investigations that have been conducted with small samples on the use of live music as an intervention for pain and anxiety.^2^ For example, Ferrer^36^ documented positive effects of live music on fear, fatigue, diastolic blood pressure, and relaxation levels among patients receiving chemotherapy. Holmes et al^37^ suggest that live music has advantages over recorded music, a conclusion cited as well in the review by Aldridge^38^ of music therapy. Live music has been shown to be beneficial for neonatal, pediatric, burn, postoperative, and palliative care populations.^33^,^39^ A recent study^40^ of live patient-preferred music resulted in greater anxiety reduction than recorded music. More such research is needed in emergency and acute care environments.

Preferential music is also becoming the standard in music interventions as studies show that choice, preference, and familiarity with music can enhance its effectiveness as an intervention and can contribute to reductions in pain and anxiety.^41^–^45^ Several terms are used for such interventions, including patient-preferred music, customized music, and patient-directed music.^46^,^47^ More recent studies have begun to look specifically at LPM as an intervention, using terms such as patient-preferred live music, live preferred music, and LPM. These studies have found significant improvements in the reduced perception of pain and anxiety, in particular.^42^,^48^ The review by Ramaswami and Silverman^5^ offered a neuroscience-based rationale for patient-preferred live music as a receptive music therapy intervention for adult medical patients. They suggest that there is ample neuroscientific evidence regarding the brain’s response to music, mostly pertaining to the reward system and dopamine release. They also offer evidence that both exposure to familiar stimuli and the act of making a choice may be neurologically reinforcing. A recent review^44^ found preferred live music was applicable for affective states, pain, nausea, and physiological measures for adult cancer and transplant patients.

Our findings are interesting and suggest that patients with moderate-intensity pain may be more likely to show quantifiable benefit from a LPM intervention. The absolute reductions in pain scores were more clinically relevant in patients with moderate initial pain intensity scores as opposed to higher or lower pain intensity scores. This finding was not likely due to differences in pain medication between groups because the morphine equivalent doses did not differ. Also, we noted that the most common reason patients provided for declining music and being excluded from the analysis was that they were in too much pain for music. These findings suggest there may be a specific range of pain in which music may be most appropriate and acceptable to patients in the ED setting. Additional research is needed to define the pain intensity levels most amenable to preferential live music intervention.

A notable finding of this study was that the LPM protocol was a useful means for obtaining a patient’s musical preference and represented a significant improvement from our prior approach. Additional testing and qualitative data collection at other sites could enhance the specificity and reliability of the instrument. In addition, the term “preference,” and the level of preference (ie, genre, artist, song) needed to qualify as a true preference need further definition in relation to hospital bedside music. Although this study marks significant advancement in defining the term LPM, further work is needed.

Live preferential music interventions in the ED have significant potential to alter the environment of care and impact patients’ perception of pain. There is ample qualitative evidence of beneficial impact. The strategies that we have developed to provide these alternative and unorthodox interventions in the ED is imminently scalable and easily disseminated. A larger, more narrowly focused multisite study with like organizations with a similar infrastructure and sophistication of arts in medicine programs may be required to demonstrate quantitative outcomes in reducing costs and supporting benefit in patient care in the acute setting. During our study, we did not experience negative impacts on the presence of LPM in these settings, and the music program costs for the two ED settings was reasonable: $63,000 per year. Future efficacy studies should also evaluate the cost-effectiveness of LPM in ED settings.

## LIMITATIONS

The study enrollment began at a period of extraordinarily high census during the flu season. Due to unexpectedly high patient volumes, the study was suspended for several reasons. Patients initially meeting enrollment criteria were triaged to locations that did not meet the required criteria for isolating the music to the individual patient. In addition, all personnel, including the musicians, were required to wear masks, making the intervention even more challenging and significantly changing the nature of the interaction. As a result, the music protocol was relocated to two satellite EDs where further challenges to enrollment occurred, specifically because fewer patients met the inclusion criteria for MSK pain. In addition, the greater efficiency, shorter length of stay, and lower acuity of the satellite facilities resulted in fewer opportunities for enrollment. Further, patient length of stay may have also impacted the length of time and exposure of patients to the intervention (most were only exposed to one song (53%) or two songs (36%), which, in turn, could be an important factor impacting outcomes. The reasons 50 patients declined the LPM intervention are unknown, but their decisions resulted in a smaller sample size than projected by the power analysis.

## CONCLUSION

Among ED patients who also received analgesic medication for acute MSK pain, we found a not statistically significant effect of more than 1 point reduction in pain intensity in both the control and live preferential music. Patients were enrolled using deception and were not informed of the study’s focus on music or their potential exposure to music. However, they were told initially that they would be part of a pain assessment study. In this context, we demonstrated that LPM interventions can be rigorously studied in some ED environments and were well-received by the patients who accepted the intervention. Future, adequately powered multisite studies are needed to demonstrate any potential quantitative benefit of LPM on patient outcomes in the ED setting.

## Supplementary Information



## Figures and Tables

**Figure:**
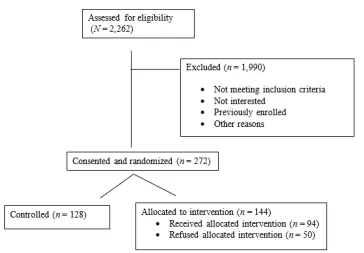
Consolidated Standards of Reporting Trials diagram showing the flow of participants through a randomized control trial of the effect of live preferential music on pain in the emergency care setting.

**Table 1:** Patient characteristics participating in a randomized control trial of live preferential music on pain in the emergency care setting.

Characteristics	Intervention (*n* = 94)	Control (*n* = 128)	Total (*n* = 222)
Age in years, mean (95% CI)	48.54 (44.63, 52.44)	48.12 (45.10, 51.15)	48.30 (45.92, 50.68)
Body mass index, mean (95% CI)	32.14 (30.20, 34.08)	30.64 (29.15, 32.13)	31.28 (30.10, 32.47)
Length of stay in hours, mean (95% CI)	4.46 (3.25, 5.67)	4.18 (3.35, 5.02)	4.4 (3.78, 5.03)
Morphine equivalents (mg), mean (95% CI)	29.49 (15.45, 43.44)	24.64 (16.55, 32.73)	26.69 (19.22, 34.16)
Sex, *n* (%)			
Male	35 (37.23)	45 (35.16)	80 (36.04)
Female	59 (62.77)	83 (64.84)	142 (63.96)
Race, *n* (%)			
White	61 (68.54)	90 (72.00)	151 (70.56)
Black	28 (31.46)	35 (28.00)	63 (29.44)
Payer status, *n* (%)			
Private/commercial	29 (32.22)	51 (41.13)	80 (37.38)
Medicare	23 (25.56)	21 (16.94)	44 (20.56)
Medicaid	24 (26.67)	32 (25.81)	56 (26.17)
Self-pay	14 (15.56)	20 (16.13)	34 (15.89)
Triage acuity, *n* (%)			
Urgent	53 (56.38)	63 (49.22)	116 (52.25)
Less urgent	39 (41.49)	58 (45.31)	97 (43.69)
Immediate	0	1 (0.78)	1 (0.45)
Emergent	2 (2.13)	6 (4.69)	8 (3.60)

Note: Other race (n = 9) are not shown here. All characteristics were non-significant at *P* < .05 level.

*CI*, confidence interval.

**Table 2:** Comparison of pain measurements in a randomized control trial of the effect of live preferential music on pain in the emergency care setting.

	Control (*n* = 128) Mean (SD)	Intervention (*n* = 94) Mean (SD)	Difference between means, (95% CI)	*P* value
Pain Now				
Baseline	6.84 (2.48)	6.28 (2.57)	−0.56 (−0.11, 1.24)	.10
Follow-up	5.63 (2.59)	4.97 (2.69)	−0.65 (−0.05, 1.36)	.07
Change score	−1.21	−1.34	−0.14 (−0.48, 0.76)	.67
Pain Worst				
Baseline	8.56 (1.74)	8.35 (1.91)	−0.21 (−0.27, 0.70)	.40
Follow-up	7.55 (2.37)	7.18 (2.54)	−0.37 (−0.28, 1.03)	.27
Change, pain intensity scores	−1.01	−1.17	−0.16 (−0.71, 0.38)	.56
Pain Least				
Baseline	4.90(3.14)	4.64 (3.02)	−0.26 (−0.57, 1.09)	.54
Follow-up	4.54 (2.83)	4.35 (2.85)	−0.19 (−0.57, 0.95)	.62
Change, pain intensity scores	−0.36	−0.29	0.07 (−0.63, 0.73)	.85

*SD*, standard deviation.

**Table 3:** Analysis of covariance model predicting follow-up pain score in a randomized control trial of live preferential music on pain in the emergency care setting.

	*t* value	*P* value	Beta coefficient
Baseline pain score	10.899	.001	0.62
Group (Intervention vs control)	−1.056	<.29	−0.306

**Table 4:** Vital signs by group and time in a randomized control trial of the effect of live preferential music on pain in the emergency care setting.

Vital signs	Control Mean (SD) *n* = 128	Intervention Mean (SD) *n* = 94
Systolic blood pressure		
Baseline	129.8 (20.13)	133.71 (24.39)
Follow-up	128.98 (19.5)	133.71 (22.05)
Diastolic blood pressure		
Baseline	75.0157 (80.13)	77.86 (13.08)
Follow-up	74.0694 (78.98)	79.46 (11.8)
Heart rate		
Baseline	74.21 (13.05)	73.36 (12.07)
Follow-up	75.94 (13.09)	75.16 (12.4)
Oxygen saturation		
Baseline	97.98 (1.91)	97.76 (1.9)
Follow-up	96.16 (10.7)	97.77 (2.13)
Respiratory rate		
Baseline	16.01 (4.5)	15.5 (1.94)
Follow-up	17.31 (10.59)	15.48 (1.83)

All differences were non-significant at *P* < .05 level.

*SD*, standard deviation.

**Table 5:** Seven themes derived from musician’s narrative reports in a randomized control trial of the effect of live preferential music on pain in the emergency care setting.

Theme	Exemplar Comments
Theme 1: Patients had positive responses to the music interaction.	“Patient said, ‘I can’t wait to come back to the emergency room!’” “Patient was very pleasant and appreciative of the music visit. She smiled and thanked me.” “Delighted afterward. Sat back and relaxed during the song, put her phone down to listen.”
Theme 2: Patients expressed gratitude for the interaction.	“She ended by saying, ‘that was a blessing,’ and I told her I was glad to be able to share that with her. She asked my name and I left with good wishes.” “Exceptionally thankful for the music and said it shifted her day.”
Theme 3: Patients reported reduction in pain.	“Patient said ‘That took the pain away.’” “Patient was really relaxed by the music. Said it helped her pain.” “He said his pain level was at a 1 when the nurse asked him. Said the music helped.” “Patient was in pain, but said the music helped.”
Theme 4: Patients participated actively in the interaction (ie, singing along, dancing).	“Praised Jesus the whole time. Sang along with each song. Looked tired but was interactive and said that God sent me.” “Sang and danced along. Seemed like it really distracted her.”
Theme 5: Initial hesitation followed by positive response to the interaction	“[Patient] was hesitant at first. Warmed up to me throughout the visit. Ended up smiling.” “Patient was hesitant, but daughter welcomed me in. I offered choices for tempo and genre and the patient smiled as I sang.”
Theme 6: The interaction provided meaningful social engagement for the patient.	“Talked a lot, about musicians they knew and other random things.” “Patient really wanted to talk about music afterwards.”
Theme 7: Patients shared the experience with others.	“[Phoned] his girlfriend to come in to listen. He invited his neighbors to listen also.” “Patient closed his eyes and sang along to all of the songs, looking over at his guest with approval.”
