# Supplementary material for: Impact of Live Preferential Music on Pain in the Emergency Care Setting: A Randomized Controlled Trial
Source: West J Emerg Med. 2026 Sep 9;27(5):1189–98. doi: 10.5811/westjem.61331 (PMC13660609; doi:10.5811/westjem.61331)
Supplement: Supplementary file 1 [file wjem-27-1189-s001.docx]

**Supplemental Material**

Live Preferential Music Protocol

**Live Preferential Music Protocol User Guide**

Upon entering a patient room, a musician will typically engage in a conversation to explore musical preference. The following guide is not intended to serve as a script, but rather, as a framework to guide this conversation and assist with documentation of preference and the general pathway taken to achieve preference.

1) To broach this conversation, start with a question like, *“What would you like to hear?”* or *“What kind of music do you like?”*
2) If the patient has an initial preference:
   a) Note and play the requested song(s)
   b) If the patient requests an artist or genre, try to get to a song-level preference **or** play something similar to the requested genre/artist, with the patient’s permission. Be sure to use the word “like,” rather than “*how about…”* or “*is it OK if I play…”*
   c) Indicate on the survey the level of preference obtained from the patient (P1-S, P2-A, P2-S, P3-G, P3-A, or P3-S)
3) If the patient has no initial preference:
   a) Ask if they have a preferred genre or artist
   b) If patient does not offer a preference, ask about tempo: *“would you prefer something relaxing or upbeat?”*
   c) Indicate on the survey the level of preference you obtained from the patient

(NP1-A, NP1-S, NP2-G, NP2-A, NP2-S, NP3-T, NP3-G, NP3-A, or NP3-S)

4) Indicate “other” for any other pathway taken, or if you are unsure of the pathway. Please provide an explanation.
5) Indicate “No preference obtained” if you were not able to achieve preference from the patient
